# Mechanisms Affecting the Gut of Preterm Infants in Enteral Feeding Trials

**DOI:** 10.3389/fnut.2017.00014

**Published:** 2017-05-08

**Authors:** Nicholas D. Embleton, Janet E. Berrington, Jon Dorling, Andrew K. Ewer, Edmund Juszczak, John A. Kirby, Christopher A. Lamb, Clare V. Lanyon, William McGuire, Christopher S. Probert, Stephen P. Rushton, Mark D. Shirley, Christopher J. Stewart, Stephen P. Cummings

**Affiliations:** ^1^Newcastle Neonatal Service, Newcastle Hospitals NHS Foundation Trust, Newcastle upon Tyne, UK; ^2^Institute of Health and Society, Newcastle University, Newcastle upon Tyne, UK; ^3^Institute of Cellular Medicine, Newcastle University, Newcastle upon Tyne, UK; ^4^School of Medicine, University of Nottingham, Nottingham, UK; ^5^College of Medical and Dental Sciences, Institute of Metabolism and Systems Research, Birmingham University, Birmingham, UK; ^6^Clinical Trials Unit, University of Oxford, Oxford, UK; ^7^Department for Biomedical Sciences, School of Life Sciences, Northumbria University, Newcastle upon Tyne, UK; ^8^Centre for Reviews and Dissemination, University of York, York, UK; ^9^Department of Cellular and Molecular Physiology, Institute of Translational Medicine, University of Liverpool, Liverpool, UK; ^10^School of Biology, Newcastle University, Newcastle upon Tyne, UK; ^11^Alkek Center for Metagenomics and Microbiome Research, Department of Molecular Virology and Microbiology, Baylor College of Medicine, Houston, TX, USA; ^12^School of Science and Engineering, Teesside University, Middlesbrough, UK

**Keywords:** lactoferrin, preterm infant, gut microbiota, metabolome, nutrition, late-onset sepsis, necrotizing enterocolitis, mechanistic evaluation

## Abstract

Large randomized controlled trials (RCTs) in preterm infants offer unique opportunities for mechanistic evaluation of the risk factors leading to serious diseases, as well as the actions of interventions designed to prevent them. Necrotizing enterocolitis (NEC) a serious inflammatory gut condition and late-onset sepsis (LOS) are common feeding and nutrition-related problems that may cause death or serious long-term morbidity and are key outcomes in two current UK National Institutes for Health Research (NIHR) trials. Speed of increasing milk feeds trial (SIFT) randomized preterm infants to different rates of increases in milk feeds with a primary outcome of survival without disability at 2 years corrected age. Enteral lactoferrin in neonates (ELFIN) randomizes infants to supplemental enteral lactoferrin or placebo with a primary outcome of LOS. This is a protocol for the mechanisms affecting the gut of preterm infants in enteral feeding trials (MAGPIE) study and is funded by the UK NIHR Efficacy and Mechanistic Evaluation programme. MAGPIE will recruit ~480 preterm infants who were enrolled in SIFT or ELFIN. Participation in MAGPIE does not change the main trial protocols and uses non-invasive sampling of stool and urine, along with any residual resected gut tissue if infants required surgery. Trial interventions may involve effects on gut microbes, metabolites (e.g., short-chain fatty acids), and aspects of host immune function. Current hypotheses suggest that NEC and/or LOS are due to a dysregulated immune system in the context of gut dysbiosis, but mechanisms have not been systematically studied within large RCTs. Microbiomic analysis will use next-generation sequencing, and metabolites will be assessed by mass spectrometry to detect volatile organic and other compounds produced by microbes or the host. We will explore differences between disease cases and controls, as well as exploring the actions of trial interventions. Impacts of this research are multiple: translation of knowledge of mechanisms promoting gut health may explain outcomes or suggest alternate strategies to improve health. Results may identify new non-invasive diagnostic or monitoring techniques, preventative or treatment strategies for NEC or LOS, or provide data useful for risk stratification in future studies. Mechanistic evaluation might be especially informative where there are not clear effects on the primary outcome (ISRCTN 12554594).

## Introduction

Preterm delivery is associated with an increased risk of mortality and serious morbidities. Among these late-onset sepsis (LOS) and necrotizing enterocolitis (NEC) are of particular concern. Adverse neurodevelopmental outcome is a major cause of long-term morbidity associated with preterm birth and along with other serious medical problems represents a major cost to health-care services and society. The UK National Institutes for Health Research (NIHR) Health Technology Assessment (HTA) programme has funded two large randomized controlled trials (RCTs) of nutrition and feeding in preterm infants born <32 weeks gestation: the speed of increasing milk feeds trial (SIFT) (ISRCTN 76463425) and the enteral lactoferrin in neonates (ELFIN) trial (ISRCTN 88261002). These trials are the largest interventional trials in preterm infants conducted in the UK and Europe with almost 5,000 proposed recruits from more than 50 neonatal units. In the planning stage, it was anticipated that infants might be eligible for recruitment to both as the timing of the trials was predicted to overlap. There was, therefore, an explicit intention to enable and support recruitment to both trials and avoid unnecessary duplication of trial processes and procedures.

In the SIFT trial, 2,804 preterm infants in the UK were randomized to one of two different rates of increase in milk feeds: increasing by 18 or 30 ml/kg/day.^1^ Recruitment commenced in August 2013 and completed ahead of schedule in June 2015. The primary outcome is survival without moderate or severe disability at 2 years corrected age, and follow-up and data collection will not complete until 2018. The SIFT protocol allowed for publication of key outcomes at hospital discharge including LOS and NEC. In the ELFIN trial, 2,200 UK infants are being randomized to either receive supplemental enteral bovine lactoferrin or placebo (sucrose).^2^ ELFIN is anticipated to complete toward the end of 2017. Both SIFT and ELFIN are pragmatic trials with key neonatal morbidities as primary outcomes, but neither included mechanistic evaluation.

This publication describes the protocol for a mechanistic evaluative study of both trials—the mechanisms affecting the gut of preterm infants in enteral feeding trails (MAGPIE) study. This will examine patterns of gut microbiota and metabolites and will be conducted in a subset of infants recruited to the main trials. Prospective recruitment to MAGPIE will only be possible for infants in the ELFIN study, although research ethics permissions exist to analyze samples from around 100 babies in the SIFT study. Participation does not affect the main trial protocols or conduct and will use safe and non-invasive collection of specimens (urine and stool) from ~480 infants from 10 sites. In addition, in those infants who undergo surgery, the MAGPIE study will aim to retrieve gut tissue from pathology archives after all necessary routine clinical tests are completed.

## Scientific Background

Prematurity is a major cause of mortality and serious long-term morbidity with an enormous burden on health care and educational systems with total costs to the public sector of approximately £UK 3 billion per year in the UK (1). NEC, a serious inflammatory bowel disease, and LOS are responsible for more deaths after the first week of life in extremely preterm infants than any other single pathology (2). NEC is associated with significant mortality (<20–40%) and affects <10% of infants born <32 weeks gestation (3–5) and occurs in first few weeks (6). In the UK, there are at least 6,000–8,000 births per year <32 weeks (7). LOS will affect <20–30% of these infants, of whom 1 in 10 may die (8). National data on cause of death in preterm infants are not routinely collected, but extrapolating data from one health region (2) suggests there are at least 250 deaths from NEC and LOS alone per year in England, although the true figure may be higher because postmortems are not always performed and clinical coding can be inaccurate. This number of deaths is similar to that for all childhood cancers (9), but unlike childhood cancer there is very little mechanistic work in preterm infants despite the worldwide rate of preterm births continuing to increase (10). NEC and LOS are both associated with significant morbidity in survivors including worse cognitive outcome and a twofold increase in the risk of cerebral palsy, and very high health-care costs: costs of surgery for NEC and prolonged intensive care are in excess of £UK 100,000 per case (5). In the US, it has been estimated that total costs related to the treatment of NEC and its consequences may be in excess of $US 5 billion per year (5). In addition, the long-term costs to society, the individual, and their families due to lifelong physical and mental impairment are substantial.

The SIFT and ELFIN trials do not include evaluation of disease pathophysiology. The practicalities of conducting trials in multiple sites over prolonged time periods means a pragmatic balance has to be reached between the detail and complexity of data collection. In SIFT, there were 56 recruiting neonatal units (primarily neonatal intensive care units, NICUs) but at least an additional 150 neonatal units required research ethics and governance approvals to collect and report trial-related data. Over 100 neonatal units who looked after SIFT babies will therefore be required to report data on the primary outcome of disability-free survival at 2 years corrected age. In the ELFIN trial, there are 35 recruiting sites (primarily NICUs) but data collection from an additional ~100 neonatal units is required in order to collect data until discharge to home. Collecting even limited biological samples from all recruited infants would be extremely challenging and expensive.

The interventions explored in SIFT and ELFIN both act *via* effects on the gut and are therefore likely to involve interactions with gut microbiota (2, 11–14). MAGPIE will use the opportunity provided by two large RCTs to explore some of the putative actions of the interventions as well as potentially explore disease mechanisms where NEC or LOS occur. MAGPIE will do this by sampling stool and urine from the infants and use emerging technologies including next-generation sequencing of gut bacteria, and urine and stool mass spectrometry, as windows into host and bacterial metabolism, respectively. The aim is to understand some of the mechanisms of actions of the interventions and diseases and provide new data in the areas of diagnosis, monitoring, and therapeutics.

### Overview of the SIFT and ELFIN Trials

Speed of increasing milk feeds trial and ELFIN trials are funded by the HTA and managed by the National Perinatal Epidemiology Unit Clinical Trials Unit, Oxford, UK.^3^ Both trials recruit very preterm infants (<32 weeks gestation) in the first few days after birth while receiving care on a neonatal unit. The trial interventions complete prior to hospital discharge, although follow-up for SIFT continues until 2 years corrected age. SIFT recruited 2,804 infants and is powered to detect a clinically important difference in disability-free survival at 2 years, as well as having adequate power to detect a difference in the rate of the key short-term outcomes NEC and LOS. Infants were enrolled when stable, tolerating some milk (but less than 30 ml/kg/day), and when the attending clinician was ready to start increasing the amount of milk feeds. Infants were randomized to increases in milk feeds of either 18 or 30 ml/kg/day. Infants from multiple pregnancies (twins, triplets, etc.) were corandomized to the same treatment arm. It was anticipated that the speed of milk feed increases would result in full feeds (defined as at least 145 ml/kg/day) being achieved about 4 days later in the slower arm. This affects the duration of central venous access and use of parenteral nutrition, although this was not mandated in the SIFT protocol and therefore is likely to affect two key risk factors for LOS. The speed of milk feed increases may also affect exposure to breast milk in the first few days of life, a further risk factor for both LOS and NEC. SIFT is a trial of comparative clinical effectiveness and recognizes the possibility that there might be competing outcomes, i.e., there may be opposing impacts on NEC and LOS between the two trial arms (15). Hence, the use of disability-free survival as an outcome that takes this possibility into account. In addition, if there are no differences in key clinical outcomes (including neurological development), then data collected for health economic analyses may determine which regime is adopted in clinical practice.

The ELFIN trial is evaluating whether supplemental bovine lactoferrin added to milk feeds affects the rate of sepsis and is powered to detect a clinically important reduction in the primary outcome (LOS) from 18 to 13% and will recruit 2,200 infants. Infants are randomized to receive either bovine lactoferrin (150 mg/kg/day) or placebo 150 mg/kg/day (sucrose) both added to milk feeds. The RCT is blinded using masked pots containing the investigational medicinal product (IMP). Unlike SIFT, infants from multiple pregnancies are randomized independently. An internal pilot phase started in June 2014 in five sites, with recruitment to the main trial commencing in July 2015. The trial is currently in progress and anticipated to complete recruitment toward the end of 2017. Further details of the trial are available (16).

### Existing Mechanistic Evaluation of NEC and LOS in a Prospective Trial Setting

Despite the major contribution of NEC and LOS to neonatal mortality and serious morbidity, there are few large interventional studies in preterm neonates exploring biological mechanisms. This is partly because of the challenges faced by adequately powered interventional studies that typically require sample sizes of >1,000 infants to detect realistic effects on NEC or LOS. Undertaking large clinical studies with mechanistic evaluation in vulnerable preterm neonates presents many complexities practically, logistically, and ethically. There are particular issues with biological sampling, especially of blood from small infants, for example, a 500-g infant has only 40 ml of total circulating blood (17). NICUs are extremely busy environments and clinical needs take precedence over activities that are purely research orientated, e.g., collection of stool samples. While NEC and LOS are major causes of morbidity, they only affect a minority of infants, with an incidence of 5–10 and 20%, respectively, in the highest risk infants (<32 weeks): a typical NICU may only admit 100–150 such infants a year. Thus, collaboration between multiple NICUs is essential for performing research with sufficient statistical power. Securing ethics and R&D approvals, collecting samples and accessing freezers close to NICUs, and storing and transporting biological samples across multiple hospital sites present many logistic challenges. In addition, the timing of onset of NEC or LOS is highly variable and unpredictable meaning that considerable “over-sampling” is required to ensure appropriate informative sampling relative to disease onset.

Perhaps because of these challenging reasons, few RCTs powered to explore differences in NEC or LOS in preterm infants have involved biological sampling on a large scale. The largest ever UK probiotic trial PiPS (3) explored whether the probiotic strain was detectable in the stool by qPCR at 2 weeks of age and 36 weeks corrected gestational age (while infants were still receiving the probiotic) and noted (a) in 11% of probiotic-treated infants the administered probiotic strain could not be detected in their stools and (b) 49% of those receiving placebo had the administered probiotic strain in their stools. Broader impact on the microbiome in this trial is awaited. These results differ from those in a large Australian probiotic trial in preterm infants [ProPrems (18)] where microbiome analysis was undertaken in 43 unblinded infants and showed (a) 8 who were in the active intervention group all carried probiotic strains and (b) only 3 out of 35 infants who were in the placebo group carried any of the probiotic strains in stool (19). Again further microbiomic impact of colonization status is awaited. Other trials have used standard culture to evaluate presence of probiotic strains in the stool of infants in an RCT, demonstrating significant differences in detection rates above or below 27 weeks gestation and significant detection in the “placebo” recipients (20, 21). None of these nested studies has to date published further microbiomics, metabolomics, or other data.

### Existing Mechanistic Evaluative Research of Feeding Rates and Supplemental Lactoferrin

Recent Cochrane systematic reviews of trials of early enteral feeding strategies for preterm infants have explored the role of trophic feeds, timing of introduction, and rate of increase in feed volume (22, 23), but none of these studies incorporated detailed mechanistic evaluation of gut function or microbiota. This might be important because alterations of gut microbes appear to be one of the key mechanisms involved in NEC or LOS pathogenesis (12, 13, 24–28). However, whether abnormal gut microbial communities are causative, or whether they simply reflect other processes involved in disease initiation is uncertain, and so can only be adequately explored in prospective trials. In the absence of a consistent causative agent in NEC studies, recent data demonstrate a specific microbial signature of high diversity and dominance of bifidobacteria that may be protective (28).

Breast milk is a complex biological fluid and differs from artificial formula milk in many respects. Components that differ include protein quality (including human lactoferrin), peptides and free amino acids, lipids (including long-chain polyunsaturated fatty acids), carbohydrates including human milk oligosaccharides (HMOs), cells, cytokines, and growth factors (e.g., insulin-like growth factor 1, epidermal growth factor, insulin, etc.). HMOs cannot be digested by the host, but rather act as growth substrates for specific *Bifidobacterium* spp. (29, 30). In the context of the SIFT trial, delayed initiation or slower increases in milk feeds reduce exposure to breast milk, which may affect gut epithelial development, function, and the pattern of gut microbiota.

At the time of funding MAGPIE, the only existing published RCT in preterm infants (*n* = 450) using bovine lactoferrin showed reductions in the incidence of LOS for a range of bacteria, both Gram-negative and Gram-positive, as well as fungi (31). The study did not include any mechanistic evaluation using biological samples. Subsequently, other studies have confirmed a reduction in LOS (32, 33), and further analyses have suggested an effect on NEC (34, 35). Recent reviews have highlighted the potential mechanisms of action for lactoferrin (36). Lactoferrin, a member of the transferrin family, is a key component of the mammalian innate immune response (37). It is the major whey protein in human colostrum and is also present in tears and other secretions, as well as being released from secondary granules in poly-morphonuclear leukocytes (38). Concentrations in human colostrum are especially high (39). However, preterm infants ingest little milk in the first few days, and this may be further inhibited by the delayed lactogenesis frequently seen in women who deliver preterm. Lactoferrin intake in preterm infants is therefore probably far lower than in healthy term neonates.

Lactoferrin has broad microbiocidal activity by mechanisms such as cell membrane disruption, iron sequestration, inhibition of microbial adhesion to host cells, and prevention of biofilm formation (16, 31, 40, 41). Development of resistance to lactoferrin is unlikely as it would require multiple simultaneous mutations. Lactoferrin remains a potent inhibitor of viruses, bacteria, fungi, and protozoa after millions of years of mammalian evolution (38). Lactoferrin has prebiotic properties, creating an environment in the gut that might promote the growth of beneficial bacteria and reduce colonization with potentially pathogenic organisms (42). It has direct intestinal immunomodulatory and anti-inflammatory actions mediated by modulating cytokine expression, mobilizing leukocytes into the circulation, and activating T-lymphocytes (37). Lactoferrin enhances proliferation and differentiation of enterocytes, closure of enteric gap junctions, and suppresses free radical activity when iron is added to milk.

Although the structure of lactoferrin is broadly similar across mammals (43), bovine lactoferrin differs from human lactoferrin, so while there are good theoretical reasons why it might have beneficial effects, the precise mechanisms of actions in preterm infants may differ or may not be present (40, 44–46). A recombinant form of lactoferrin [talactoferrin (TLf)] was also available in the US and has been used within an RCT as prophylaxis against infection, showing a reduction in fecal staphylococci load to almost undetectable levels in infants receiving TLf, and an associated reduction in coagulase-negative staphylococci infections. The authors also demonstrated a TLf-modulated reduction in fecal Enterobacteriaceae postulating that this may be a possible mechanism for a reduction in NEC since proteobacteria have been associated with NEC development (47).

### Recent Studies of NEC and LOS Using Microbiomic and Metabolite Data

Necrotizing enterocolitis and LOS are complex multifactorial diseases. In particular, a pathological finding of NEC is likely to represent the final common pathway of a wide array of pathophysiological processes (48–51). Recent studies show that NEC and LOS are associated with abnormal gut microbial patterns including lack of diversity, presence of “marker” bacteria, and alterations in bacterial community structures (24, 28, 52, 53). However, a key feature of most recent publications is the lack of a specific or consistent gut microbiomic signature between studies. Since 2011, when the use of next-generation sequencing came to the forefront of microbiome research, specific bacterial taxa have been associated with NEC onset, particularly those from the Proteobacteria phylum such as *Enterobacter, Escherichia, Sphingomonas*, and *Klebsiella* spp., although the studies produce different findings and many have limited power due to their small size.

However, even large studies exploring NEC produce inconsistent findings. In a single NICU in Denmark *n* = 163 preterm infants were studied (21 with NEC) and 482 samples analyzed: there were no clear differences between NEC and control infants, although Gram-positive bacteria appeared more common in NEC cases using culture, a result not confirmed in molecular analyses (54). In one of the largest studies to date, Warner et al. recruited *n* = 166 infants (46 with NEC) and analyzed 3,587 stools and found increased relative abundance of Gamma-proteobacteria and reduced Negativicutes (48). Independent of specific bacteria, it has also been shown that the overall load of bacteria (52) or the presence of fungi have no clear association with NEC onset (24, 52). Thus, while the microbiome is undoubtedly important to the pathogenesis of NEC, a specific microbe is unlikely to be causative. This is in accordance with a recent microbiome and metabolome investigation by Stewart et al., where instability of the microbiome and a lack of bifidobacteria were significant risk factors for NEC and for the generation of NEC-associated metabolites (28). Indeed, metabolites associated with NEC were not correlated to any specific bacteria, but were negatively correlated with *Bifidobacterium*. To this end, several recent papers have used multi-omic technologies to go beyond “who is there” and determine the microbe–host interaction and overall functional profiles. These studies support the notion that different microbiome communities can yield more comparability at the protein and metabolite level, demonstrating the promise for mass spectrometry-based techniques (55–57).

#### Methodological Issues Associated with Microbiomic and Metabolite Analytical Platforms

In exploratory studies, the use of two independent small molecule (metabolite) profiling techniques such as liquid chromatography mass spectrometry (LCMS) and gas chromatography mass spectrometry (GCMS) may improve the detection of metabolites of interest. GCMS will detect volatile organic compounds (VOCs) that appear to have important consequences for preterm gut development and maturation such as short-chain fatty acids (SCFAs), and the GCMS protocol we will employ in MAGPIE has been extensively optimized to ensure appropriate capture of VOCs. Likewise, it is important to employ an LCMS method optimized for the high-throughput processing of preterm samples (28, 57, 58). While GCMS methods are facilitated by spectral reference databases for the identifications of compounds, LCMS requires standard compounds to be ran to confirm otherwise putative identifications from databases that are typically based on mass to charge. While validated tools exist for the identification of metabolites in untargeted LCMS experiments such as Mummichog (59), where appropriate and available matching to standard references to confirm identifications is important. In addition, the choices of columns (LC) and fibers (GC) can have profound effects on the metabolite profiles and the detected compounds, so it is important that the methods used are validated in these sample types. Recent validation work identifying peroxidation metabolites in urine from preterm infants provides a good example of this approach (60, 61).

Furthermore, the 16S rRNA gene sequencing has been extensively applied by several groups to preterm stool research as previously described. While this technique will only allow classification of bacterial hits to genus level, it has proven to be an effective technology for microbial ecology-based research. Tools such as Tax4Fun (62) and PICRUSt (63) also exist for predicting the bacterial metagenome based on the bacteria identified. While this inference is predicted, such tools offer an important means of investigating the functional capacity of the microbiome, which can be further linked to metabolomic data. Therefore, combining these different approaches may allow investigators to discern “who is there” and “what they are doing (microbe and host).”

However, all of the above techniques have limitations, and there are many other factors that are not explored with these methods, including genomic, epigenetic, transcriptomic, proteomic, and posttranscriptional modification of proteins. However, there is already strong data to show that the approaches we aim to use in MAGPIE will provide important data. Differences in the presence of VOCs have already been linked with the development of NEC (64) and may also relate to the emergence of LOS (65, 66). In addition, organisms causing blood culture-positive sepsis in preterm infants were frequently detected within the gut prior to LOS onset, and typically as an abundant member of the gut microbial community (24).

The MAGPIE aims to explore potential interactions between microbes and metabolites critical for development of gut immune function expanding work from our group and others (67, 68). Additional studies highlight the importance of host–microbe interactions by demonstrating the pivotal role of SCFAs and other compounds in inducing differentiation of gut regulatory T cells (69–71), a pathway of major importance in preterm infant NEC and LOS.

The MAGPIE may provide insights into the effects of early gut microbe/host interactions in establishing gut health and immune function. We will build on our existing work that has optimized techniques to understand gut inflammation in the presence of suppressed or dysregulated immune systems, e.g., inflammatory bowel disease (72). These will utilize specialized immunohistochemical analysis of resected diseased and non-affected tissue and use computer-aided learning techniques, digital quantification of bright field chromogenic staining to explore implicated causal biological pathways, especially if there are differences between trial arms in SIFT and ELFIN. We will compare diseased cases with controls either recruited to the main trials or from recent cohorts in our hospitals and examine cell surface markers and cytokines that might link microbial and metabolomic changes, as well as exploring the potential for transcriptomic analysis.

## Aims and Objectives

The aim of the MAGPIE study is to explore differences in gut microbiota and metabolic correlates between trial intervention arms (feed rate, lactoferrin) and dynamic changes in the period preceding disease onset (NEC or LOS). We aim to determine effects in both the stool and urinary metabolome, because these may reflect changes in either bacterial or host metabolism, or both. Specifically, we will determine changes in the bacterial community and overall metabolome profiles using both LCMS and GCMS, and using computational tools will determine correlations between the microbiome such as predicted gene orthologs and resulting metabolite compounds. Our specific aims are to test the following hypotheses:

1. Trial interventions will result in detectable differences in gut microbiota that will be directly related to metabolic function;
2. Infants who develop NEC or LOS will have differences in gut microbiota and metabolic profile in the period preceding disease onset compared to control infants;
3. There will be detectable differences in gut tissue inflammatory response between surgically resected gut tissue affected by NEC and control tissue.

We will achieve these aims by determining the following outcomes:

1. Gut microbial diversity (e.g., Shannon Diversity Index) and differences in the proportions of key bacterial taxa measured using 16S next-generation sequencing in stool samples collected after enrollment on days 1–3, 7, 10–14, and 21 (±1) days.
2. The association between gut microbiota and the stool metabolome using mixed-effect models, structural equation modeling (SEM), and ordination analyses. Stool metabolome is measured using GCMS and/or LCMS in samples collected on days 1–3, 7, 10–14, and 21 (±1) days.
3. Pattern of gut microbiota prior to the onset of NEC or LOS measured using up to seven daily stool samples in the period immediately prior to disease compared to samples from control cases who do not develop disease.
4. The gut tissue inflammatory response in surgically resected gut tissue affected by NEC and in control tissue (either non-affected tissue from the same infant or tissue from an infant requiring gut resection who does not have disease) will be determined by immunohistochemistry. This will be measured after trial completion by retrieving samples from hospital pathology archives.

Our analytical models will also explore the effects of clinical risk factors for NEC and LOS such as gestation and markers of illness severity, and the effects of exposure to interventions such as breast milk and antibiotics, and consider other key outcomes such as time to full feeds, age at discharge, and growth, e.g., predischarge weight gain.

## Procedures—Summary

We will

1. Recruit at least 480 infants from up to 10 neonatal units in the UK enrolling infants to ELFIN.
2. Identify stored samples from infants recruited to SIFT (one site only).
3. Collect a daily stool and urine sample from MAGPIE infants until hospital discharge (average duration 40–50 days) or transfer back to local neonatal unit.
4. Retrieve any residual resected gut tissue of enrolled infants who undergo intestinal surgery.
5. Identify samples which are the most:
  - *informative*—based on trial intervention and disease presence;
  - *comprehensive*—consistency of daily sampling;
  - *representative*—balanced for trial intervention and other key factors, e.g., gestation, breast milk exposure.
6. Samples to be analyzed will include
  - *all diseased cases*: infants who meet the SIFT and ELFIN internationally agreed predefined case definitions of confirmed NEC or LOS following review at Blinded Endpoint Review Committee;
  - *non-disease cases*: infants who do not develop NEC or LOS, selected using matching algorithms to ensure trial intervention and risk factor coverage.
7. The samples analyzed will focus on the early postnatal period when trial intervention differences will be greatest: e.g., day 0—3, 6–8, 9–11, 13–15, and 20–22 in ~25–50 infants per trial intervention arm. While we aim for daily sampling we recognize this will not always be achieved due to the complexity of the NICU working environment, combined with the fact that many sick infants do not pass a stool every day. We will also analyze samples at additional time points as necessary to ensure a daily sample is analyzed for up to 7 days before diagnosis in all diseased cases and match these to non-disease controls.
8. Analyze samples for
  - *gut microbiota* using next-generation sequencing on the MiSeq (Illumina) platform to determine gut microbial patterns;
  - *stool VOC* using “headspace” GCMS;
  - *stool metabolomic profile* using LCMS to determine patterns in the metabolome;
  - *urine metabolomic profile* using LCMS and assays for inflammatory proteins, e.g., intestinal fatty acid-binding protein (iFABP) where sufficient samples and informative cases exist.
9. Determine changes due to trial interventions and changes preceding disease onset. We will explore dynamic changes in the gut community structure: proportions of key bacterial operational taxonomy units, presence of specific pathogenic strains, diversity, richness, and stability of communities.
10. Analyze resected gut tissue using optimized immunohistochemistry to determine gut immune response where NEC develops. If sufficient samples exist, explore how trial interventions (feed rate, lactoferrin) and changes in gut microbiota or metabolome may relate to histological findings.
11. Store residual samples in a Human Tissue Act (UK) and research ethics approved Newcastle University Biomedicine Biobank (the “Great North Neonatal Biobank”) for use in future studies.^4^ HTA license no. 12534, Ethics approval 15/NE/0334, IRAS 161883.

### Standard Operating Procedures (SOPs)—Sample Collection, Storage, and Transport

Samples will be collected, anonymized, and analyzed according to established SOPs already developed by members of the project team. In brief, stool samples are collected from the nappy/diaper using a clean disposable plastic spoon and placed in a glass pot with a lid. Samples will be collected at routine clinical nursing care times. Urine samples are collected according to standard NICU procedure, which typically involves collecting urine passed spontaneously onto sterile cotton wool ball, squeezed out using a sterile syringe, and aliquoted into two 2 ml cryovials. Samples are labeled and placed in a −20°C freezer (generally immediately or within an hour of collection) located on the NICU. Samples are then transferred frozen in batches from local hospitals to central laboratories where they will be stored at −80°C prior to microbiomic and metabolomic analyses. Transport of samples will take place every 6–8 weeks meaning the duration of local storage at −20°C is around 1–8 weeks, on average 3–4 weeks. We will record sample storage duration at −20 and −80°C and conduct analysis throughout the study period in a chronological fashion so that we can adjust, if required, for any confounding introduced by varying storage durations.

Short-term storage of stool samples does not significantly affect the microbial communities, although measurement of storage time of samples is important to avoid potential bias (73–75). We aim to account for the amount of time in storage for DNA extractions to minimize bias. To determine any storage effects, we will conduct quality control by comparing gold standard immediate DNA extracts from <10 samples to the same number following storage of stool for 12 and 18–24 months.

Any gut tissue resected during surgery for either NEC or other conditions (e.g., spontaneous intestinal perforation) will be retrieved from paraffin blocks located in NHS pathology archives after all clinical tests have been complete. Samples will be transported to the central laboratory for immunohistochemical analyses. It is possible that only 10–12 infants undergo surgery for NEC (or other conditions) and have residual tissue available. We will therefore consider the use of control tissue from other preterm infants available locally within one hospital (Newcastle Hospitals NHS Foundation Trust) where we have research ethics permission to analyze historical samples.

### Enrollment, Consent, and Data Collection

Parents can be approached for written informed consent at any time after enrollment to ELFIN is complete, but this will usually be within the first 3 days, and frequently will occur at the same time as consent to ELFIN. Written information will explicitly state the intention to share and use data collected for ELFIN. Research ethics approval allows the collection of stool and urine samples and storage on the NICU prior to signed consent for MAGPIE, but any such samples will be destroyed if consent is not obtained. We will use data collected for SIFT and ELFIN by electronic encrypted data transfer from the clinical trials unit (NPEU) and supplement this with additional items for MAGPIE that include specific antibiotic type, use of prophylactic antifungal and probiotic strain/brand if these are used, and date and time of stool and urine collection.

### Case Definitions of Disease (NEC or LOS)

We will use the internationally accepted definitions used by SIFT and ELFIN, which are subsequently confirmed at Blinded Endpoint Review Committees conducted by at least two senior clinician investigators blinded to trial interventions at the NPEU.

#### Late-Onset Sepsis

Microbiological culture of potentially pathogenic bacteria (including coagulase-negative staphylococci species but excluding probable skin contaminants) or fungi from fluid sampled aseptically more than 72 h after birth from blood or CSF, or clinically suspected sepsis (meeting three objective clinical criteria) AND intention for treatment for 5 or more days with intravenous antibiotics. If the infant died, was discharged, or was transferred prior to the completion of 5 days of intravenous antibiotics, this condition would still be met if the intention was to treat for 5 or more days.

#### Necrotizing Enterocolitis

Necrotizing enterocolitis may be diagnosed at surgery, at postmortem or clinically and radiologically: at least one of the following clinical signs present: bilious gastric aspirate or emesis, abdominal distension, or occult or gross blood in stool AND at least one of the following radiological features: pneumatosis intestinalis, hepatobiliary gas, or pneumoperitoneum. Infants who satisfy the definition of NEC but at surgery or postmortem have a “focal gastrointestinal perforation” will not be coded as having NEC.

All diseased cases meeting the predefined case definition of NEC or LOS will have samples analyzed (expected total ~70–100 infants). Samples from up to 200 non-diseased cases will be selected to ensure sufficient coverage of intervention arms using matching algorithms and coverage of other clinical risk factors and outcomes. We will also analyze samples from non-diseased cases to determine microbiomic and metabolomic profiles between trials arms.

### Laboratory Procedures—Stool and Urine

We will analyze bacterial DNA extracted from stool samples using our well-established protocols and 16S ribosomal RNA methods that are effective tools to explore the diversity of bacterial communities (24, 76, 77). We will conduct metabolomic profiling of stool and urine. Extraction of samples will be optimized for detection of SCFAs and samples processed using non-targeted and targeted high-resolution LCMS to generate metabolomic profiles that may indicate functional changes in the host and the gut microbiota. Our targeted approach will investigate known gut flora fermented products of complex carbohydrates, including SCFAs, acetates, amino acids, and carbohydrate fragments, which may be present in stool samples. Analysis of the stool metabolome reflects changes in gut microbial activity and may impact on host gut function such as changes in permeability. Our non-targeted approach will allow us to compare metabolomic differences between trial arms in order to define a metabolite pattern associated with sample groups that can be correlated with information on microbial diversity, health, or disease.

We have optimized a LCMS method based on C18 reverse phase chromatography coupled to a Q-Exactive high-resolution mass spectrometer validated on 100 mg of stool and has demonstrated the robust and reproducible detection of 10^3^ metabolites (28, 58). Identification of significant metabolites will be based on data-dependent tandem MS/MS and confirmed using standards. Analysis (see Statistical Analysis) will use modeling techniques to explore relationships between the microbiome and metabolome, and study interventions and disease. Examination of the urinary metabolome is more reflective of changes to the overall host (infant) metabolic state but may also reflect differences in absorption of compounds from the gut. Determining the metabolomic profiles between and within patients to complement the stool microbiome sequencing data will enable exploration of how the host, gut microbes, trial interventions, and other clinical factors may interact, and any downstream functional effects such as feed tolerance or growth. To supplement the metabolomic data, we will store samples so future studies can analyze urine and stool samples using assays for proteins such as calprotectin and iFABP that may provide additional diagnostic or mechanistic information (78–81).

Volatile organic compounds from stool samples will be analyzed by GCMS using well-established protocols for extracting and analyzing headspace gases. These methods are based on a CARB/PDMS SPME fiber, a Combipal sampler with a Peltier-cooler, and a Perkin Elmer Clarus 600 Gas Chromatograph with Clarus 600T Mass Spectrometer (82). The method is validated on as little as 50 mg of sample: sufficient to analyze ~40 compounds, which includes 8 different acids, particularly SCFAs, branched and linear, alcohols, and esters. Interpretation of fragment patterns will be undertaken against the current mass spectral NIST library, followed by manual visual inspection (82).

### Laboratory Procedures—Resected Gut Tissue

The MAGPIE study provides a unique opportunity to explore potential gut actions of feeding rate and lactoferrin in a small number of cases if NEC occurs. This may include immunomodulatory and anti-inflammatory actions mediated by modulating cytokine expression, mobilizing leukocytes into the circulation, and activating T-lymphocytes. Breast milk and lactoferrin enhance proliferation and differentiation of enterocytes and closure of enteric gap junctions (36). Tissue-based analyses will take place in two domains: (1) exploring the aberrant innate and adaptive immune mechanisms and (2) validating immune pathways or biomarkers identified by microbiomic or metabolomic profiling.

Immunohistochemistry will be performed using paraffin blocks cut into 4 µm sections for staining using a Discovery XT auto-stainer (Ventana Medical Systems, Inc., Tucson, AZ, USA). Slide images will be acquired using the Vectra 3.0 Automated Quantitative Pathology Imaging System (Perkin Elmer, Waltham, MA, USA), using techniques optimized by our group at Newcastle. Antigens of interest will be quantified digitally. Leukocyte infiltrates associated with NEC will be identified using antibodies to several epitopes, for example, markers of inflammatory cell subsets, cellular proliferation, cytotoxic granule expression, transcription factors, or cytokines. Many of these antibodies have been previously optimized for intestinal tissue by the team at Newcastle, and new antibodies will be optimized as required informed by data generated in other work strands of this project. Staining of “healthy” resection margins from the same patient will be undertaken where possible and control analyses will be performed by use of matched non-NEC control tissue collected from MAGPIE infants, supplemented if needed by further samples from the Newcastle upon Tyne Hospitals biorepository (e.g., cases of spontaneous neonatal perforation). Targeted exploratory histology will validate immune mechanisms highlighted by metabolomics or microbial assays, and *in situ* hybridization may also be performed to identify transcription of relevant immune pathways identified by VOC, microbiomic, or metabolomic profiling.

### Sample Size Estimates

Sample size requirements for each element of this study have been evaluated and are based on both published data and practical/logistical aspects from our existing work and that from other published studies. These considerations include

1. The duration, estimated start time, and recruitment rate for ELFIN, along with the estimated start date for MAGPIE and logistical issues in establishing recruitment from 10 hospital sites.
2. The trial efficacy and disease event rate will not be known until the main trial completes. The incidence of NEC (Bell stage 2 or greater) is expected to be 5–10% and for LOS <20%, and some infants will have both diseases. Recruiting 480 infants will identify between 70 and 100 “disease” cases, 10–20 cases requiring gut surgery, and provide well sampled “non-diseased” infants with varying clinical risk factors, exposures, and outcomes.
3. VOC analysis may identify several individual compounds; in our recent paper publishing the methodology, we show that on average 31.3 ± 10.5 (mean and SD) VOCs were identified per sample (82). At a power of 80%, and two-sided significance at the 5% level, we would need 50 infants per trial intervention group to show an increase in 5 VOCs. The SD for each sample was 2.9 ± 1.3 compounds and on average 90% of the VOC abundances showed a coefficient of variation smaller than 30%. Our data also showed in a study of 13 infants, which VOC number in healthy neonates significantly increased with age (0.49 extra VOCs per day 95% CI 0.12–0.86), a trend not seen in those who developed NEC (64).
4. Microbiomic data complexities means that the sample size necessary to evaluate the actions of different interventions and the incidence of disease is dependent on effect size, the number of interacting factors, and their correlation. For a power of 80% to detect a 50% difference in community profile patterns arising from a categorical descriptor of microbial community variation, using a two-sided test at a significance level of 5%, the study needs ~200 samples. In our previous studies (1) 12 twin pairs analyzed (gut microbial profiling) using PLS-DA showed highly significant correlations between pairs (53) and (2) examination of 136 samples from 32 patients (*n* = 20 NEC or LOS) showed significant differences (*p* = 0.002) in microbiomic patterns between diseased and healthy individuals (24).
5. Using immunohistochemical data from our group (83), and assuming that any differences between disease and control will be greater than those in healthy individuals, and using a two-sample *t*-test, our proposed sample size of 20 would give 80% power to detect a difference of 0.66 (66%) in cells/crypt cross section at a significance level of 5% and an approximated SD of 0.5 (50%).
6. Mixed-effects models, which we propose for modeling the direct effects of the microbiome on disease risk, are economical with power because the residual variance of these models is smaller since some of it is accounted for in the random effect. Using the method by Cohen (84), we calculate that a GLM would have a power of 82% with 10 predictors and 50 for a “large” effect size (*f*^2^ = 0.35, *R*^2^ = 0.51) at the 5% level of significance, and a power of 47% for a “medium” effect size (*f*^2^ = 0.15, *R*^2^ = 0.36) at the 5% level of significance. With *n* = 100 the calculated powers are 99% for a large effect size and 74% for a medium effect size. Including random effects in the GLM would increase power at the same effect size or conversely permit smaller effect sizes at the same power. The power analysis of an SEM is altogether more complex because it relies on the goodness-of-fit criteria selected for the model. Power at the model level may be low when there are few model degrees of freedom even for a reasonably large sample size; requiring greater than 100 samples if there are fewer than 20 model degrees of freedom (85). We will supplement these models where needed using a Bayesian SEM approach.

### Statistical Analysis

There are three major modeling issues that have to be addressed in this analytical pathway:

(i) the multivariate nature of the microbiome and metabolome, and data generated from immunohistochemistry;
(ii) the longitudinal/developmental component in the neonate, which will lead to repeated measures of disease state and microbiome on the same individuals, and
(iii) the interdependence and interactions between different predictors, which may have both direct and indirect effects on incidence and progression of disease.

The analytical approach used in this study will focus on modeling the relationship between putative risk factors, trial interventions, and incidence of NEC and LOS from longitudinal data capturing variation in both risk factors and disease through time, and the any differences in non-diseased cases between trial arms. We will use a progressive modeling strategy based on combining multivariate analyses of micro- and metabolomic immunological data, with mixed-effect modeling of outcome in relation to drivers of disease; and finally, mixed-effect SEM to quantify the importance of interacting factors and drivers. Specifically, we will

(i) quantify the impacts of individual risk factors in causing disease;
(ii) quantify the effects of direct and indirect risk factors on disease;
(iii) quantify the dynamics of pathogens, the neonatal microbiome, the immune response, and their impact on outcomes, and
(iv) identify the impacts of trial interventions and clinical management on the drivers and disease.

## Anticipated Results from Analysis of Microbiome and Metabolome

We will use multivariate ordination techniques to summarize and visualize the major trends in variation in microbial community composition of infants’ stool collected. The analyses will identify those taxa most closely associated with microbiome change through time. We will use canonical ordination to quantify the impacts of other covariates (diet, age, and interventions) on the microbiome composition. We will identify key microbial taxa in the ordination space that capture the trend in variation in relation to disease and use these with the results of the ordination as input variables for the subsequent mixed-effect and SEM analyses below. To ensure the results are not spurious, the observed differences in metabolomic profiles will be validated by permutation testing in MetaboAnalyst 3.0 (86). Where appropriate, significance of categorical variables were determined using the non-parametric Mann–Whitney test for two category comparisons or the Kruskal–Wallis test when comparing three or more categories (87) and all *p*-values were adjusted for multiple comparisons with the false discovery rate algorithm (88).

MixOmics (89) will be implemented to determine the correlation between the relative abundance of the dominant bacterial taxa from 16S rRNA gene sequencing and the intensity of metabolites of interest by sparse partial least squares regression (90). Longitudinal and network analyses will facilitate investigations into the directionality of any observations. Tax4Fun (62) will be used to infer the metabolic potential of the microbiome based on the 16S data. Model-based integration of metabolite observations and species abundances will then be applied to determine biologically feasible correlations between the inferred bacterial KEGG orthology and the resulting metabolites of interest (91). This will determine which metabolites are likely to be bacterial derived. It is important to note that in the course of data generation, new tools are likely to emerge that can perform distinct analyses on multi-omic data. Where appropriate we will incorporate such tools where they are likely to (1) outperform existing tools and/or (2) allow novel analyses to be performed.

### Quantifying the Direct and Indirect Impact of Risk Factors on Disease

We will use repeated measure mixed-effect modeling to quantify the direct effects of microbiome on risk of disease while adjusting for clinical factors. We will use case as the random effect and adjust for autocorrelation in the response using appropriate correlation structures in the model. The applicants have used this approach extensively to investigate the epidemiology of food-borne pathogens (92). We will use an SEM approach to quantify direct and indirect effects of risk factors on the incidence of disease. We will develop a set of models that characterize the relationship between clinical factors, the changes in gut microbial community types, developmental stage (postnatal and postmenstrual age), and comorbidities associated with disease onset and then challenge the model with the data derived from the laboratory and clinical data collection (93).

### Plan for Interim Statistical Analyses

The MAGPIE study funder (NIHR) and the project team agreed that it would be appropriate to conduct a futility-type analysis to ensure there are measurable impacts on the microbiome after ~150 (of the total planned 480) infants have been recruited, and prior to commencing further analysis of the microbiome and metabolome. The ELFIN study is blinded so any laboratory and statistical analysis of anonymized samples while recruitment continues will be conducted by investigators blinded to trial interventions. Blinding to trial intervention arm will also be maintained while gut tissue is examined.

For the interim analyses we will

1. Select samples taken before disease onset at around 7–10 days after commencing the trial IMP (lactoferrin or placebo) in up to 60 infants per trial group so as to compare and assess microbial community changes between trial groups. Because other risk factors are also associated with disease (e.g., gestation, postnatal age, etc.), we will use progressive model building that investigates microbial community dynamics in relation to these covariates.
2. Use multivariate ordination and classification approaches to quantify trial intervention group differences in gut microbial communities. This will (i) quantify the variation in microbial community composition before disease onset and (ii) develop suitable covariates describing microbial community variation for inclusion in statistical analysis as risk factors for disease.
3. Use unconstrained ordination to investigate trends in community composition and to identify those microbial taxa from the NGS data that contribute most to community variation. These axes may be used as covariates in the subsequent analysis.
4. Use constrained ordination to investigate the impact of trial intervention group on community variation prior to disease onset.
5. Use divisive classification approaches to identify classes of microbial community among the cases. This will create a suite of categorical descriptors of community composition. Higher level community descriptors (diversity, evenness, rate of change in ordination score) will be derived in case the overall structure of the community is an important driver.
6. In the interim analyses, we will determine whether there is any effect on the total microbial community differences or on individual bacterial taxa using MANOVA and consider a difference in the microbial community of *p* < 0.05 to be proof an effect. If the *p* value is between 0.05 and 0.1, we will consider this to be strong evidence of a likely effect, where statistical significance might subsequently be achieved in an analysis with larger numbers and/or greater numbers of diseased or high risk cases and will complete the study as planned. Regression models and SEM will adjust for potential confounding variables such as age, sex, gestation, and feed and antibiotic exposures.

## Summary

The MAGPIE study provides a unique opportunity to explore the interaction between nutritional and feeding interventions on gut microbiota in early life in preterm infants, and within the context of large randomized trials. Next-generation sequencing and metabolomic techniques produce large datasets that require complex modeling in order to present findings of clinical relevance. While these new “omic” techniques have provided unique insights into complex multifactorial diseases such as NEC, current studies are limited by their observational nature and are at significant risk from type I errors due to residual confounding and/or reverse causation. Prospective trials are needed to determine direct causation due to clinical interventions such as milk feed rate or enteral lactoferrin supplementation. Nesting mechanistic evaluation within these trials using non-invasive and safe sampling increases the value of large RCTs. This may produce important new data of diagnostic and therapeutic relevance or identify differences in responses between groups of infants (i.e., exploring in what setting interventions may be most or least efficacious), as well as potentially providing information about risk stratification for future studies. NEC and LOS remain extremely prevalent and serious diseases in preterm infants and both are associated with considerable mortality and morbidity, as well as generating significant health-care resource costs. Studies such as MAGPIE will improve our understanding of the basic biology of health and disease and the role of early life gut microbiota colonization in a high risk group of patients where mechanistic understanding is limited.

## Ethics Statement

This study received research ethics approval from East Midlands—Nottingham 2 Research Ethics Committee 16/EM/0042.

## Author Contributions

NE, JB, JD, AE, EJ, JK, CAL, CVL, WM, CP, SR, MS, CS, and SC all made substantial contributions to the study design, drafted and/or revised this manuscript, and approved it for publication. All authors agreed to be responsible for its integrity.

## Conflict of Interest Statement

This work was supported by the EME programme 13/122/02. None of the authors have any relevant financial relationships, patents, copyrights, or royalties relevant to this submitted work. NE has received support for other research studies from manufacturers of infant milk formula and also received speaker fees from manufacturers of parenteral nutrition products designed for preterm infants.

## Footnotes

^1^www.npeu.ox.ac.uk/sift.

^2^www.npeu.ox.ac.uk/elfin.

^3^https://npeu.ox.ac.uk/trials.

^4^http://www.ncl.ac.uk/nbb/.

## Abbreviations

ELFIN, enteral lactoferrin in neonates trial; GCMS, gas chromatography mass spectrometry; HMOs, human milk oligosaccharides; iFABP, intestinal fatty acid-binding protein; LCMS, liquid chromatography mass spectrometry; LOS, late-onset sepsis; NEC, necrotizing enterocolitis; NICU, neonatal intensive care unit; NIHR, National Institutes for Health Research; PN, parenteral nutrition; RCT, randomized controlled trial; SCFA, short-chain fatty acids; SEM, structural equation modeling; SIFT, speed of increasing milk feeds trial; TLf, talactoferrin (recombinant); VOC, volatile organic compound.
